# Effect of Donor Age on the Proportion of Mesenchymal Stem Cells Derived from Anterior Cruciate Ligaments

**DOI:** 10.1371/journal.pone.0117224

**Published:** 2015-03-02

**Authors:** Dae-Hee Lee, Joanne Ng, Sang-Beom Kim, Chung Hee Sonn, Kyung-Mi Lee, Seung-Beom Han

**Affiliations:** 1 Department of Orthopaedic Surgery, Samsung Medical Center, Sungkyunkwan University School of Medicine, Seoul, Korea; 2 Department of Biochemistry and Molecular Biology, Division of Brain Korea 21 Program for Biomedical Science, Korea University College of Medicine, Seoul, Korea; 3 Barunmadi Orthopaedics, Seongnam, Gyeonggi-do, Korea; 4 Department of Orthopaedic Surgery, Korea University College of Medicine, Seoul, Korea; University of Wisconsin-Madison, UNITED STATES

## Abstract

The characteristics of anterior cruciate ligament (ACL)-derived mesenchymal stem cells (MSCs), such as proportion and multilineage potential, can be affected by donor age. However, the qualitative and quantitative features of ACL MSCs isolated from younger and older individuals have not yet been compared directly. This study assessed the phenotypic and functional differences in ACL-MSCs isolated from younger and older donors and evaluated the correlation between ACL-MSC proportion and donor age. Torn ACL remnants were harvested from 36 patients undergoing ACL reconstruction (young: 29.67 ± 10.92 years) and 33 undergoing TKA (old: 67.96 ± 5.22 years) and the proportion of their MSCs were measured. The mean proportion of MSCs was slightly higher in older ACL samples of the TKA group than of the younger ACL reconstruction group (19.69 ± 8.57% vs. 15.33 ± 7.49%, p = 0.024), but the proportions of MSCs at passages 1 and 2 were similar. MSCs from both groups possessed comparable multilineage potentiality, as they could be differentiated into adipocytes, osteocytes, and chondrocytes at similar level. No significant correlations were observed between patient age and MSC proportions at passages 0–2 or between age and MSC proportion in both the ACL reconstruction and TKA groups. Multiple linear regression analysis found no significant predictor of MSC proportion including donor age for each passage. Microarray analysis identified several genes that were differentially regulated in ACL-MSCs from old TKA patients compared to young ACL reconstruction patients. Genes of interest encode components of the extracellular matrix (ECM) and may thus play a crucial role in modulating tissue homeostasis, remodeling, and repair in response to damage or disease. In conclusion, the proportion of freshly isolated ACL-MSC was higher in elderly TKA patients than in younger patients with ACL tears, but their phenotypic and multilineage potential were comparable.

## Introduction

Mesenchymal stem cells (MSCs) have the properties of self-renewal, high proliferative capacity and inherent multilineage differentiation potential, suggesting their possible use for tissue regeneration or repair of damaged tissue[1,2]. While bone marrow is a good source of MSCs[3], these cells can also be isolated from other adult mesenchymal tissues, including fat[4], trabecular bone[5], synovium[6], synovial fluid[7], and anterior cruciate ligament (ACL)[8]. As the torn ACL is usually discarded during ACL reconstruction, and degenerative ACL is removed during total knee arthroplasty (TKA), these removed tissues can be a potential source of MSCs.

Because the generation of multipotent ACL-derived MSCs capable of differentiating into target cells in a reproducible and controlled manner is critical to clinical success[9], the quality and purity of MSCs isolated from ACL tissues *ex vivo* are important. Despite the potential use of ACL-derived MSCs (ACL-MSC) in clinical applications, especially for providing cell sources for ligament and tendon reconstruction, little is known about the *ex vivo* properties of ACL-MSCs. Moreover, since the ages of patients who undergo ACL reconstruction and those who undergo TKA differ markedly, it is important to evaluate the effect of donor age on the proportion and multilineage potential of MSCs isolated from ACL tissue. To date, however, the qualitative and quantitative features of ACL-MSCs isolated from younger and older patients have not been compared directly. This study was therefore designed to assess the phenotypic and functional differences in ACL-MSCs isolated from younger and older donors, as well as the correlation between proportion of ACL-MSCs isolated and donor age. We hypothesized that the proportion of MSCs in ACL samples are higher in younger patients undergoing ACL reconstruction than in older patients undergoing TKA, and that the proportion of MSCs correlates negatively with donor age.

To further define differences between ACL-MSCs from TKA and ACL reconstruction patients, we compared the gene expression profiles of these MSCs using microarray analysis. Microarray analyses have been used to evaluate gene expression profiles in MSCs derived from various sources, such as synovium[10,11], meniscus, intraarticular ligament[12], bone marrow[13], umbilical cord[14], and adipose tissue[15]. These studies suggest that genes participating in various biological, immunological and metabolic processes could be differentially regulated for stem cell survival, growth and development. The gene expression patterns of MSCs could reveal additional information on the effect of age on MSC properties and provide clues to improve the tissue regeneration potential of these MSCs for therapeutic applications.

## Materials and Methods

### Ethics Statement

The ethical approval of this study protocol was granted by Institutional Review Board of the Korea Univerisity Anam Hospital (permit no. AN09041). Written informed consent was obtained from all subjects before participation in this study (parental/guardian consent was obtained for minors).

### Study design and collection of ACL samples

This prospective study enrolled all candidates for TKA due to severe osteoarthritis, and all candidates for ACL reconstruction due to isolated ACL ruptures, as confirmed by magnetic resonance imaging (MRI) and physical examinations, such as positive anterior drawer, Lachmann, and/or pivot shift tests (more than grade II). Patients with diagnoses other than osteoarthritis (e.g. pyogenic arthritis, rheumatoid arthritis, or traumatic meniscal tears), and those undergoing revisional TKA, were excluded. Patients with other concomitant intra-articular (e.g. meniscus or ligament injuries) or associated extra-articular lesions, and those undergoing revisional ACL reconstruction, were also excluded to exclude any effects of other potential sources of MSCs on the proportion of MSCs in ACL grafts. ACL samples were easily recovered from patients undergoing TKA, because the entire ACL was resected. In patients undergoing ACL reconstruction, torn ACL remnants were harvested with pituitary forceps.

### Cell isolation and culture

For cell isolation, the same areas (mid-substances) of the ligament in each patient were used to obtain P0 cells. The synovial sheath and fat tissues were carefully removed to obtain only the ACL fascicles. The ACL samples were washed twice with 1X phosphate buffered saline (PBS), minced into small pieces, and incubated with 3% collagenase type I (Worthington Biochemical Corporation, Lakewood, NJ, USA) in 1X PBS at 37°C. After 2 hours, the digested samples were washed with 1X PBS, centrifuged at 1500 rpm for 10 min, and the supernatants were aspirated. The pellets were reconstituted in 50 ml 1X PBS and filtered through a 70-μm nylon cell strainer (BD Biosciences, Franklin Lakes, NJ, USA), using a 15-mL syringe plunger to remove debris. The suspensions were centrifuged at 1500 rpm for 10 min and the supernatants were discarded. Red blood cells were lysed by adding 1 ml ACK lysing buffer (150 mM NH_4_Cl, 10 mM KHCO_3_, 0.1 mM Na_2_EDTA, pH 7.2–7.4) for 5 min at room temperature (RT). The tube was topped with 1X PBS, and the cells were filtered through a nylon mesh, centrifuged at 1500 rpm for 5 min, and resuspended in complete Poietics Mesenchymal Stem Cell Growth Medium (MSCGM) (Lonza Walkersville, Inc., Walkersville, MD, USA).

### Cell expansion

1 × 10^5^ primary ACL-derived MSCs were cultured for 14 days in 10 mL of MSCGM and incubated in a humidified 5% CO_2_ incubator at 37°C. Then, cells were detached with 1 mM ethylenediaminetetraacetic acid (EDTA) in PBS and counted using Trypan Blue (Sigma, St. Louis, MO, USA). 1 × 10^5^ P1 cells were seeded into a 100-mm diameter culture dish containing 10 mL of MSCGM, incubated in 37°C, 5% CO2 incubator, and harvested after 7 days to get P2 cells[16,17].

### Flow cytometry

**Fig 1:**
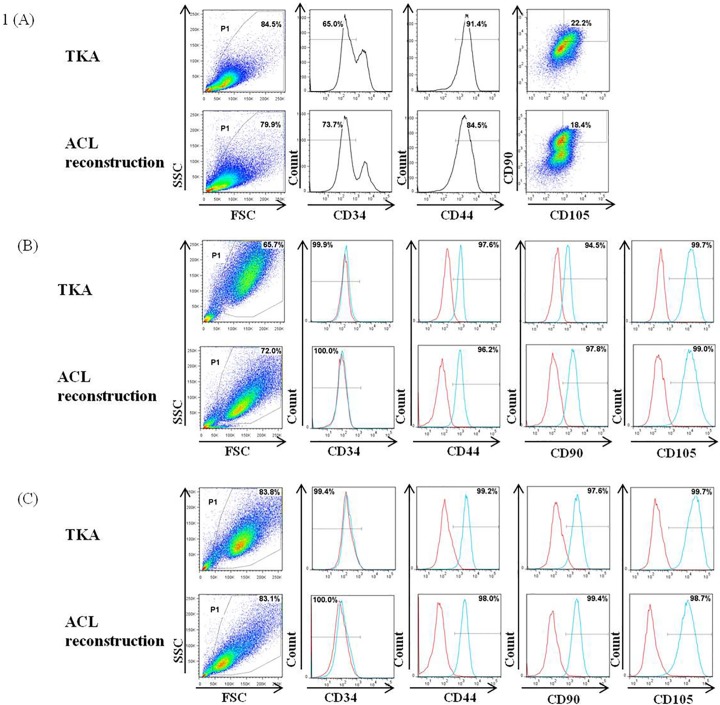
Immunophenotype of human anterior cruciate ligament (ACL)-derived mesenchymal stem cells (MSCs) from total knee arthroplasty (TKA) and ACL reconstruction. Cells from TKA and ACL reconstruction samples at (A) passage 0, (B) passage 1 and (C) passage 2 were stained with antibodies against CD34, CD44, CD90 and CD105 and analyzed by flow cytometry. CD34 negative populations were gated in live populations (P1); CD44 positive populations were regated in CD34 negative populations; CD90 and CD105 positive populations were regated in CD44 positive populations. Representative histograms are shown in blue lines while red lines represent isotype controls.

Flow cytometry assays were performed to characterize the proportion of ACL-derived MSCs in passage 0, 1, and 2 cells. Cultured cells were harvested with 1 mM EDTA in PBS, centrifuged at 1500 rpm for 5 min, and resuspended in 1 mL MSCGM. 1 x 10^5^ cells were transferred to a polystyrene round-bottom tube (BD Biosciences), centrifuged at 1500 rpm for 3 min, and resuspended in 100 μL of FACS buffer containing monoclonal antibody (mAb). After incubation for 20 min at 4°C, the cells were washed with 1 mL FACS buffer and fixed in 300 μL of 1% paraformaldehyde in PBS. Fifty thousand cells per sample were acquired and analyzed using a FACSCanto II instrument (BD Biosciences) and FlowJo. The primary antibodies included fluorescein isothiocyanate-conjugated antihuman CD34 mAb, peridinin chlorophyll protein-cyanine 5.5 (PerCP-Cy5.5)-conjugated antihuman CD90 mAb, and allophycocyanin (APC)-conjugated antihuman CD105 (endoglin) mAb, purchased from eBioscience (San Diego, CA, USA); and phycoerythrin-conjugated antihuman CD44 mAb from BD Biosciences. MSCs were defined as cells triple positive for CD44, CD90 and CD105 and negative for CD34. Acquired cells were gated as P1 and surface expression of CD44, CD90, and CD105 was assessed in CD34–populations (Fig. 1).

### Confirmation of MSC multipotentiality

For adipogenesis, 1.9 x 10^4^ cells at passage 1 or 2 were plated in a 24 well-plate and cultured in 1 mL MSCGM. The medium was then switched to 1 mL complete STEMPRO adipogenesis differentiation medium (Invitrogen, Carlsbad, CA, USA) once cells were 100% confluent. Cells were maintained in adipogenic medium for 2 weeks with medium changes twice weekly. The adipogenic cultures were fixed in 10% formalin (Sigma-Aldrich, St. Louis, MO, USA) for 1 h at RT and stained with fresh Oil Red O solution (stock: 0.3% in isopropanol, mixed three parts stock to two parts water and filtered through a 0.2 m filter; Sigma-Aldrich) for 1 h at RT. Cells were then washed with water until the washes ran clear. Cells were visualized with a light microscope and photographed. To quantify adipogenic differentiation, the Oil Red O stain was eluted by adding 100% isopropanol (Sigma-Aldrich) for 10 min at RT. Absorbance at 490 nm was read in triplicates.

For osteogenesis, 0.95 x 10^4^ cells at passage 1 or 2 were plated in a 24 well-plate and cultured in 1 mL MSCGM. The medium was replaced with 1 mL complete STEMPRO osteogenesis differentiation medium (Invitrogen) once cells were 50–70% confluent. Cells were maintained in osteogenic medium for 2 weeks with medium changes twice per week. The osteogenic cultures were fixed in 1 mL ice cold 70% ethanol (Sigma-Aldrich) for 1 h at 4°C and stained with 4mM Alizarin Red S in distilled water (pH adjusted to 4.2 with ammonium hydroxide; Sigma-Aldrich) for 10 min at RT. The excess dye was removed and washed four times with water. Cells were photographed with a light microscope. To quantify osteogenic differentiation, 400 mL of 10% (vol/vol) acetic acid was added to each well and incubated for 30 min with shaking. The cells were gently scraped with a cell scrapper and transferred with 10% (vol/vol) acetic acid (Sigma-Aldrich) to a 1.5-mL microcentrifuge tube. The tube was sealed with parafilm, vortexed vigorously for 30 seconds, heated to 85°C for 10 min and then transferred to ice for 5 min. After centrifugation at 20,000 g for 15 min, the supernatant was transferred to a new 1.5-mL microcentrifuge tube. The pH was adjusted to 4.1–4.5 with 10% (vol/vol) ammonium hydroxide (Sigma-Aldrich). Absorbance at 415 nm was read in triplicates.

For chondrogenesis, 1.65 x 10^5^ cells at passage 1 or 2 were placed in a 15-mL conical tube and centrifuged at 1500 rpm for 5 min. The pellets were cultured in 0.5 mL of complete STEMPRO chondrogenesis differentiation medium (Invitrogen) for 3 weeks with medium changes twice weekly. Photographs of the pellets were taken with a ruler for size analysis. The pellets were fixed in 4% paraformaldehyde for up to 2 days and then placed in 1 mL 30% sucrose at 4°C for 1 day. Cryosections (10 μm) were mounted onto slides and stained with Toluidine Blue O (Sigma-Aldrich). Photographs were taken with a light microscope. To quantify chondrogenic differentiation, pellets were fixed with 4% paraformaldehyde for 15 min, washed twice with 1X PBS, and stained with Toluidine Blue O for 15 min. The cells were washed again with 1X PBS to remove the unbound dye. The dye was extracted with 1% SDS and the absorbance at 595nm was read in triplicates.

### Microarray analysis

Total RNA was isolated from second-passage cells using Trizol (Invitrogen) and purified using RNeasy columns (Qiagen, Valencia, USA) according to the instructions of the manufacturers. The RNA samples were treated with DNase 1 and subjected to additional clean-up procedures. Then, the RNA samples were quantified, aliquoted and stored at -80°C until use. Quality control was performed using denaturing gel electrophoresis, OD 260/280 ratio, and Agilent 2100 Bioanalyzer (Agilent Technologies, Palo Alto, USA). Biotinylated cRNA samples were prepared from 550ng of total RNA using the Ambion Illumina RNA amplification kit (Ambion, Austin, USA) according to the manufacturer’s protocol. Then, 750 ng of labeled cRNA samples were hybridized to each human HT-12 expression v.4 bead array according to the manufacturer's instructions (Illumina, Inc., San Diego, USA). The array signal was detected using Amersham fluorolink streptavidin-Cy3 (GE Healthcare Bio-Sciences, Little Chalfont, UK) according to the bead array manual. Arrays were scanned with an Illumina bead array Reader confocal scanner according to the manufacturer's protocol. Raw data were extracted using the Illumina GenomeStudio v2011.1 (Gene Expression Module v1.9.0) software. For data analysis, array probes with detection p-value ≥ 0.05 (similar to signal to noise) in over 50% samples were filtered out. Selected gene signal value was transformed by logarithm and normalized by quantile method. Fold change was used to evaluate the statistical significance of the expression data. Hierarchical cluster analysis was done using complete linkage and Euclidean distance as a measure of similarity. Gene-Enrichment and Functional Annotation analysis for significant probe list was conducted using DAVID (http://david.abcc.ncifcrf.gov/home.jsp). All data analysis and visualization of differentially expressed genes was performed using R 2.15.1 (http://www.r-project.org/). All microarray data have been deposited in the Gene Expression Omnibus (GEO) database (accession number GSE59662).

### Statistical analysis

Mean differences in MSC populations (i.e., cells positive for CD44, CD90 and CD105 and negative for CD34) in samples from patients who underwent TKA and those who underwent ACL reconstruction were evaluated using Student’s t-test for parametric data and the Mann-Whitney U-test for non-parametric data. Repeated measures analysis of variance (ANOVA) was also used to compare differences between MSC sources (TKA vs. ACL reconstruction) and culture time (passages 0, 1, and 2). Although the significance level was *a priori* p = 0.05 for all statistical tests, the level of significance was corrected using the Tukey method when multiple comparisons were performed.

Correlations between patient age and the proportions of MSCs in the three passages were assessed by Pearson correlation analysis. Multiple linear regression analysis was performed to identify variables that independently affected MSC proportion at each passage. The results were also checked for multicollinearity by examining the variance inflation factors (VIFs) of the predictor variables. If one variable has a VIF >10, this indicates multicollinearity and the variable should be removed from the multiple linear regression analysis due to the possibility of it being a confounding factor. In multiple linear regression analyses, female gender was coded as 0 and male gender as 1. A p value <0.05 was considered statistically significant.

## Results

### Patient demographic data, proportion of mesenchymal stem cells, and multipotentialities

Of the 78 patients (78 knees) initially approached for the study, 75 agreed to take part. After eligibility assessments, 73 patients were enrolled (38 in the ACL reconstruction group and 35 in the TKA group). Two patients in the ACL reconstruction group were excluded because unexpected longitudinal meniscal tears, not visible on MRI, were detected during surgery. Two patients in the TKA group were also excluded because the ACL was absent or worn out. Thus, the final analysis included 36 patients who underwent ACL reconstruction and 33 who underwent TKA. These two groups differed significantly in age and gender distribution, but not in body mass index (BMI; Table 1). Apart from the differences in age, as TKA are typically performed in elderly female subjects, a majority of patients in the TKA group were female (84%) while those in the ACL reconstruction group were male (72%).

**Table 1:** Demographic characteristics of patients with (sub)acute and chronic ACL tears.

	ACL Reconstruction group	Total Knee Arthroplasty group	*P*-value
Number of patients	36	33	
Gender (Male: Female)	26: 10	5: 28	<0.001
Age (years), mean ± SD	29.67 ± 10.92	67.96 ± 5.22	<0.001
Body mass index (kg/m^2^), mean ± SD	25.18 ± 3.28	27.73 ± 4.03	0.474

At passage 0, the mean proportion of MSCs was significantly higher in ACL cells from the TKA than from the ACL reconstruction group (19.69 ± 8.57% vs. 15.33 ± 7.49%, p = 0.024). However, MSC proportions at passages 1 and 2 were similar in the two groups (Table 2).

**Table 2:** MSC proportion at each passage in the ACL reconstruction and total knee arthroplasty groups.

	ACL Reconstruction group (mean ± SD)	Total Knee Arthroplasty group (mean ± SD)	p-value
**Passage 0 (%)**	15.33 ± 7.49	19.69 ± 8.57	**0.024**
Passage 1 (%)	74.12 ± 12.98	73.16 ± 8.97	0.721
Passage 2 (%)	78.56 ± 11.48	75.44 ± 7.81	0.190

MSC, mesenchymal stem cell; ACL, anterior cruciate ligament; SD, standard deviation

Boldface text indicates a significant between group difference in mean MSC proportion.

**Fig 2:**
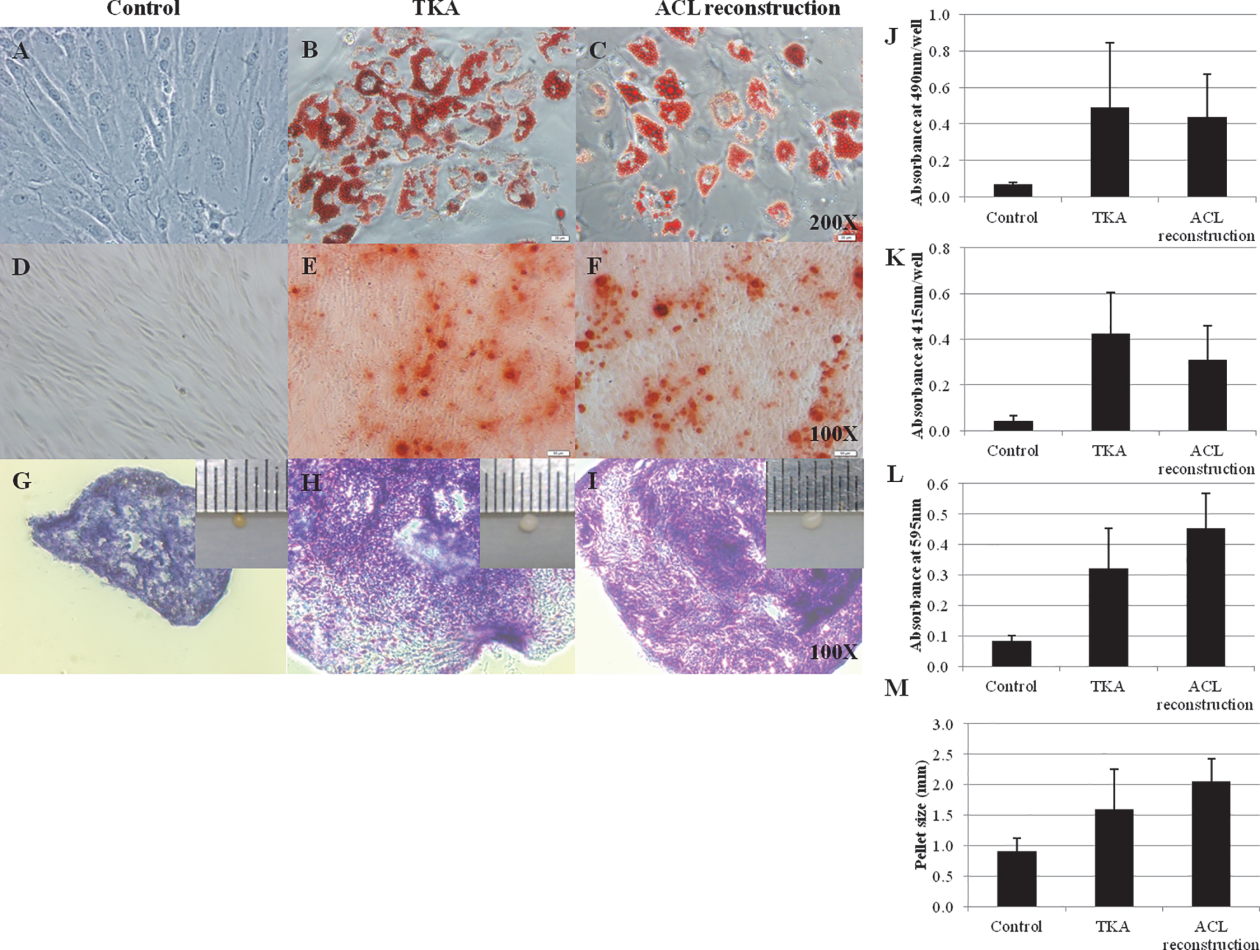
Adipogenic, osteogenic, and chondrogenic potentials of TKA and ACL reconstruction cultures *in vitro*. (A) Cells were cultured in control medium (A, D, G), adipogenic medium (B, C), osteogenic medium (E, F) or chondrogenic medium (H, I). Adipogenesis was evaluated by staining cells with Oil Red O and quantifying absorbance at 490nm (J). Osteogenesis was evaluated by staining cells with Alizarin Red S and quantifying absorbance at 415nm (K). Chondrogenesis was evaluated by staining cells with Toluidine Blue O and quantifying absorbance at 595nm (L). Chondrogenic pellet size was measured with a 1-mm scaled ruler (M). All data shown were the results of n≥3 per group.

We found that the proportion of ACL-MSCs in cell cultures from both groups increased markedly with serial passage, from passage 0 to 2 (p < 0.001), but the rate of increase from initial isolation to passage 2 was similar in both groups (p = 0.505). To investigate the multilineage potentiality of these ACL-derived MSCs, cells at passage 1 or 2 were cultured in adipogenic, osteogenic, and chondrogenic conditioned media. MSCs from both groups could be successfully differentiated into adipocytes, osteocytes, and chondrocytes (Fig. 2 A-I).

To determine the adipogenic potential of ACL-MSCs from TKA and ACL reconstruction groups, we stained adipogenic cultures with Oil Red O, extracted the dye with isopropanol and measured absorbance at 490 nm. Oil Red O staining showed that lipid vacuoles accumulated in cells induced with adipogenic medium in both TKA and ACL reconstruction groups. However, there were no significant differences of absorbance at 490 nm between TKA and ACL reconstruction groups (0.49 ± 0.36 versus 0.44 ± 0.23, p = 0.875, Fig. 2J).

To evaluate the osteogenic potential, we stained osteogenic cultures with Alizarin Red S, extracted the dye and measured absorbance at 415 nm. Alizarin Red S staining showed calcifications in cells induced with osteogenic medium in both groups. Similarly, there were no significant differences of absorbance at 415 nm between TKA and ACL reconstruction groups (0.42 ± 0.18 versus 0.31 ± 0.15, p = 0.547, Fig. 2K)

To compare the chondrogenic potential of ACL-MSCs from TKA and ACL reconstruction groups, we measured the size of the resulting chondrogenic pellets and performed histological staining with Toluidine Blue O. After 3 weeks of culture with chondrogenic medium, cell pellets from both groups became spherical and developed a glistening, transparent appearance. The development of the extracellular matrices of chondrogenic pellets were shown by staining with Toluidine Blue O. In addition, there were no significant differences of absorbance at 595nm between TKA and ACL reconstruction groups (0.32 ± 0.13 versus 0.45 ± 0.11, p = 0.258, Fig. 2L). Comparison of chondrogenic pellet sizes showed that there were also no significant differences between ACL-MSCs from TKA and ACL reconstruction groups (1.60 ± 0.65 mm versus 2.10 ± 0.37 mm, p = 0.121, Fig. 2M). Taken together, these data demonstrate that ACL-MSCs from TKA and ACL reconstruction groups have similar adipogenic, osteogenic and chondrogenic potential.

### Correlations and predictors of MSC proportions

We observed no significant correlation between patient age and proportion of MSCs at passages 0, 1, and 2. In addition, age was not correlated with MSC proportion in each group individually (Table 3).

**Table 3:** Correlation between age and MSC proportion at each passage.

		Age of overall patients	Age of ACL reconstruction group	Age of TKA group
Passage 0	*r*	0.156	0.254	0.099
	*P*-Value	0.197	0.119	0.584
Passage 1	*r*	-0.155	-0.316	0.173
	*P*-Value	0.201	0.181	0.336
Passage 2	*r*	-0.136	-0.174	0.323
	*P*-Value	0.263	0.661	0.167

*r*, Pearson correlation coefficient; ACL, anterior cruciate ligament; TKA, total knee arthroplasty.

Multiple linear regression analyses were performed to identify predictors of MSC proportion at passages 0, 1, and 2. Age, gender, and BMI were included as potentially predictive variables of MSC proportion at each passage. Multiple linear regression analysis across all patients, as well as in each group individually, showed no significant predictors of MSC proportion at each passage (Table 4). All independent variables have a VIF of less than 1.5, Rj2<0.3, indicating that there was no association between the independent variables.

**Table 4:** Multiple linear regression analysis of predictors of the proportion of ACL-MSCs at passage 0 in overall patients and in the ACL reconstruction and total knee arthroplasty groups.

*Dependentvariables*	*Independent Variables*	*Unstandardized coefficients*		*Standardized coefficients*		
		*B*	*SE (B)*	*β*	*P-value*	*VIF*
ACL-MSC P0 (%) of overall subjects	Age	0.018	0.056	0.046	0.750	1.423
	Gender (male)	-2.762	2.317	-0.167	0.238	1.374
	Body mass index	0.172	0.272	0.080	0.529	1.119
ACL-MSC P0 (%) of ACL reconstruction group	Age	-0.301	0.101	-0.445	0.065	1.034
	Gender (male)	-2.358	2.546	-0.144	0.361	1.120
	Body mass index	0.402	0.343	0.178	0.251	1.085
ACL-MSC P0 (%) of TKA group	Age	0.175	0.291	0.107	0.552	1.007
	Gender (male)	7.135	4.671	0.276	0.137	1.043
	Body mass index	-0.352	0.385	-0.166	0.368	1.050

P, passage; MSC, mesenchymal stem cell; ACL, anterior cruciate ligament

B, unstandardized coefficients; SE (B), standard error of B; β standardized coefficients

The adjusted R^2^ for ACL-MSC proportion at passage 0 were 0.053 in all patients, 0.228 in the ACL reconstruction group, and 0.095 in the TKA group.

### Microarray analysis

**Fig 3:**
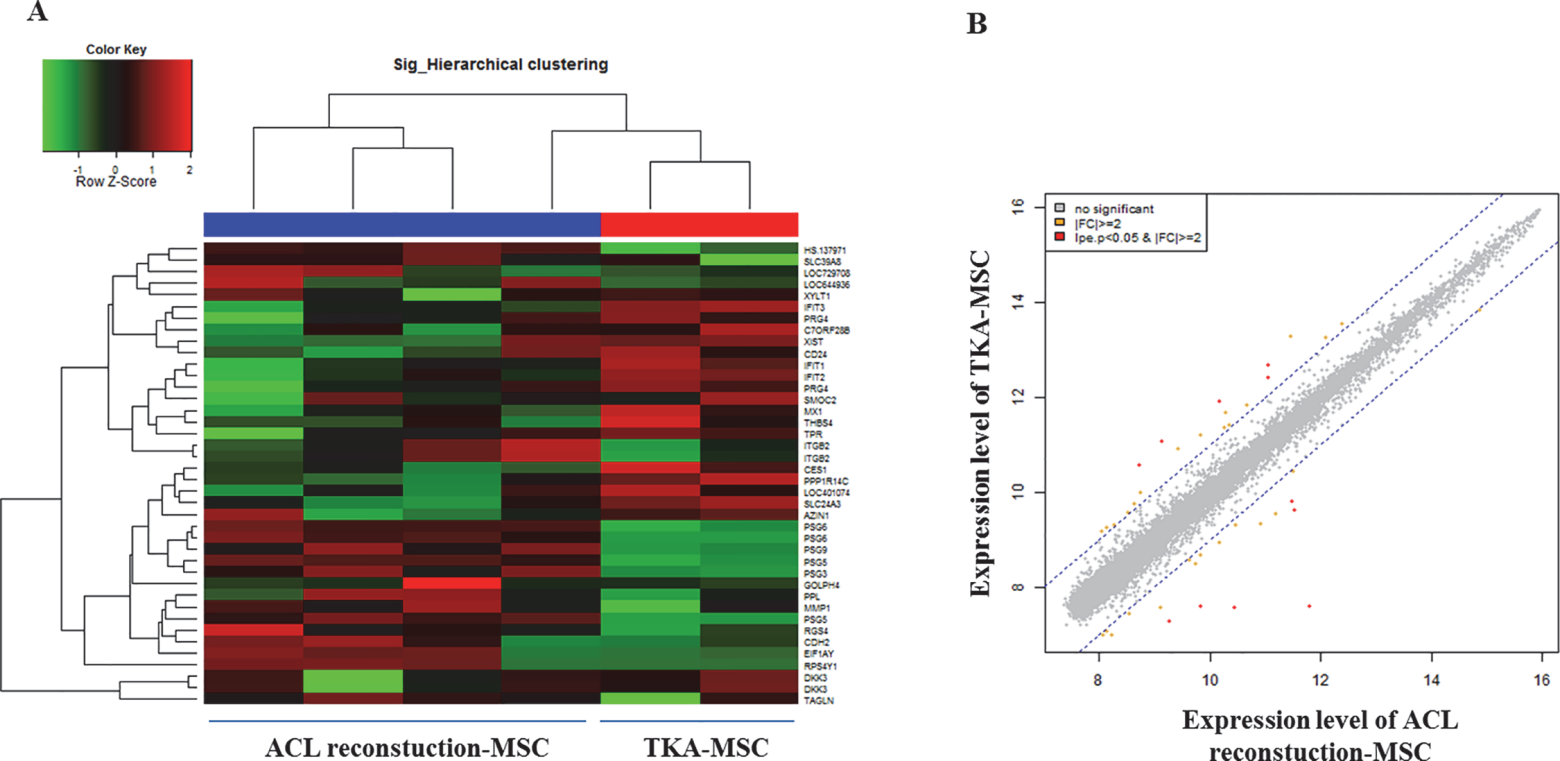
Microarray analysis of ACL-derived MSCs from the ACL reconstruction and total knee arthroplasty (TKA) groups. (a) Hierarchical clustering of the total of 47,320 ACL-MSC transcripts in both groups. Red indicates upregulation and green downregulation. (b) Scatter plots showing the log ratios of the means of transcripts in ACL-derived MSCs from the TKA relative to the ACL reconstruction group. Red dots indicate a >2-fold induction (p < 0.05).

To determine the gene expression patterns of ACL-MSCs from the two patient groups, microarray analysis of samples at passage 2 was performed. Gene profiles of passage 2 MSCs isolated from patients who underwent ACL reconstruction and those who underwent TKA are shown in Fig. 3A,B. There were 40 genes that showed 2- to 18-fold differential regulation in the TKA and ACL patients analyzed. These included genes that coded for molecules involved in various biological processes such as, cell communication and adhesion (27.5%), cell structure and motility (5%), protein metabolism and modification (15%), immunity and defense (12.5%), and transport (5%). Other genes were non-coding (5%), coded for hypothetical proteins (2.5%) and proteins with unclassified molecular functions and biological processes (27.5%). Among these, we chose to highlight only genes that had a fold change of more than 2 and a p-value of less than 0.05. The top three genes showing higher expression in MSCs from the TKA than from the ACL reconstruction group (Table 5) were C7orf28B (chromosome 7 open reading frame 28B), XIST (X (inactive)-specific transcript (non-protein coding)) and PRG4 (proteoglycan 4), whereas the top 3 genes showing reduced expression in ACL-MSCs from the TKA than from the ACL reconstruction were RPS4Y1 (ribosomal protein S4), PSG5 (pregnancy specific beta-1-glycoprotein 5), and EIF1AY (eukaryotic translation initiation factor 1A).

**Table 5:** Upregulation and downregulation of genes in TKA-MSCs compared with ACL-MSCs.

Ref Seq NM	Gene Symbol	Fold change	P-value	DEFINITION
NM_198097.1	C7orf28B	3.848	0.00001	Chromosome 7 open reading frame 28B (C7orf28B)
NR_001564.1	XIST	3.706	0.00000	X (inactive)-specific transcript (non-protein coding) (XIST)
NM_005807.3	PRG4	3.398	0.00930	Proteoglycan 4 (PRG4), transcript variant A
NM_002462.2	MX1	3.113	0.00276	Myxovirus (influenza virus) resistance 1, interferon-inducible protein p78 (mouse) (MX1)
NM_003248.3	THBS4	2.594	0.00403	Thrombospondin 4 (THBS4)
NM_001008.3	RPS4Y1	-18.121	0.00000	Ribosomal protein S4, Y-linked 1 (RPS4Y1)
NM_002781.2	PSG5	-7.116	0.00000	Pregnancy specific beta-1-glycoprotein 5 (PSG5)
NM_004681.2	EIF1AY	-4.658	0.00000	Eukaryotic translation initiation factor 1A, Y-linked (EIF1AY)
NM_002781.2	PSG5	-3.866	0.00222	Pregnancy specific beta-1-glycoprotein 5 (PSG5)
NM_000211.1	ITGB2	-3.653	0.00403	Integrin, beta 2 (antigen CD18 (p95), lymphocyte function-associated antigen 1; macrophage antigen 1 (mac-1) beta subunit) (ITGB2)
NM_000211.2	ITGB2	-3.105	0.03896	Integrin, beta 2 (complement component 3 receptor 3 and 4 subunit) (ITGB2)

Positive values represent upregulation. Negative values represent downregulation.

## Discussion

We compared the proportion and expandability of ACL-MSCs in older patients who underwent TKA and younger patients who underwent ACL reconstruction to assess the effects of donor age on the properties of ACL-MSCs. Contrary to our expectations, the principal findings of this study indicated that the proportion of freshly isolated ACL-MSCs was higher in the elderly TKA group than in the young ACL reconstruction group.

It remains unclear whether MSCs from older patients lack the abilities to expand and differentiate. Although several studies found that age had no effect on the properties of bone-marrow-derived MSCs, including colony-forming ability, adipogenesis, and calcification[18], other studies have reported that age affected cell proliferative capacity at passage 1, as well as the chondrogenic, osteogenic, and adipogenic differentiation ability of bone-marrow-derived MSCs[19]. No obvious differences were observed in the yield or colony-forming efficiency of freshly isolated cells at passage 0 between bone-marrow-derived MSCs from younger and older donors[20]. Moreover, a recent study comparing the characteristics of MSCs derived from the synovium and subcutaneous fat found no notable age-associated differences in proliferative ability at passage 1 or multipotentiality[21]. To our knowledge, our study was the first to evaluate the impact of donor age on the proportion of ACL-derived MSCs. We found no significant differences in ACL MSC populations between younger patients undergoing ACL reconstruction and older patients undergoing TKA. Previously reported discrepancies on the effects of aging on MSC biological activity may be attributable to donor-related factors other than age, variations in culture conditions and/or MSC tissue sources, sampling site (bone marrow or ACL), and/or methods of isolating and/or quantifying MSC.

In contrast to our hypothesis, we found that aging had no effect on the proportion of ACL-MSCs. Rather, the MSC proportions at passage 0 were higher in older patients undergoing TKA than in younger patients undergoing ACL reconstruction. Several hypotheses can explain these results. For example, although disruption and loss of articular cartilage are hallmarks of knee osteoarthritis, this disease leads to degenerative and destructive changes in other intra-articular soft tissues, such as the synovium, meniscus, and ligament[22]. MSCs in synovial fluid of osteoarthritic knees may originate from intra-articular soft tissue, including the ACL. These MSCs are thought to take part in tissue homeostasis, remodeling, and repair of damaged intra-articular structures in osteoarthritic knees. The severe osteoarthritis of patients in our TKA group may give rise to an increased number of MSCs with regenerative capacity, thus explaining, at least in part, the lack of aging effect on the proportion of ACL derived MSCs.

MSC characteristics can also be influenced by their peri-cellular environment as well as by the health of the organ in which they reside, suggesting that tissue cells may effectively influence the fate of MSCs and MSC derivatives[23]. Therefore, rupturing of the ACL in younger patients undergoing ACL reconstruction may affect the intra-articular environment, causing the release of MSCs from ruptured ACL fibers and changing “replicative cell senescence”. Finally, mild, undetectable inflammation in osteoarthritic knees may affect the proportion of ACL derived MSCs[11]. A postmortem study of 120 knee joints from patients aged 23 to 92 years found a positive correlation between ACL sheath inflammation and donor age, and between sheath inflammation and damage to cartilage[24]. Emerging evidence suggests that MSCs may contribute more to the repair of damaged tissue through the secretion of soluble factors that induce local cell regeneration than by direct induction of stem cell differentiation[25]. This paracrine effect may be due to an increase in the number of MSCs in the local environment where inflammation occurs.

Interestingly, we found that five genes were upregulated and six genes were downregulated in ACL-MSC samples from older patients undergoing TKA compared with those from younger patients. The relationship of most of these genes to ACL-MSCs from TKA and ACL reconstruction patients remains unclear; however, knowledge of the differential gene regulation could be helpful to clarify the mechanisms of tissue homeostasis, remodeling and repair modulated by MSCs in different age groups. Among the genes more highly expressed in ACL-MSCs from TKA patients compared to those from ACL reconstruction patients was the gene encoding proteoglycan 4 (PRG4). PRG4, or lubricin, is a component of the cartilage extracellular matrix and synovial fluid which participates in the lubrication of joints[26]. Since lubricin has been shown to protect against both age-related and post-traumatic osteoarthritis[27], it is surprising that in our study, PRG4 was found to be upregulated in patients who had developed osteoarthritis. This may represent a compensatory mechanism in TKA-MSC samples against osteoarthritis and could, in part, explain why the MSC proportion was higher in the TKA group. In addition, the expression of lubricin has been shown to be regulated by inflammatory cytokines such as IL-1, TNF-α, TGF-β and BMP-7[28–30]. In both a rat model and in humans, reduced levels of lubricin in the synovial fluid has been linked with ACL rupture-associated joint inflammation[31,32]. This indicates another possibility that the inflammatory cascade could have mediated downregulation of lubricin after ACL rupture in ACL reconstruction patients. Another gene that was upregulated in ACL-MSCs from TKA patients compared to ACL reconstruction patients was thrombospondin 4 (THBS4). THBS4 encodes a component of the ECM in tendons and ligaments which mediates cell-to-cell and cell-to-matrix interactions within the ECM[33,34]. Lack of THBS4 was shown to negatively affect the organization, composition and functions of tendons and muscles, indicating that THBS4 was an important regulator of the ECM composition in these tissues[35]. The higher expression of THBS4 in TKA patients was also unexpected but it does lend support to our finding that the MSC proportion was higher in TKA patients compared to ACL reconstruction patients. It is possible that the expression of THBS4 in TKA patients was increased for better ECM remodeling in response to tissue damage. Interestingly, integrin beta 2 (ITGB2), a receptor for THBS4, was downregulated in ACL-MSCs of TKA patients. Studies have shown that ITGB2 interacted with THBS4 in the regulation of inflammation[36,37]. Furthermore, synovial membrane tissues from dogs with cranial cruciate ligament rupture showed increased levels of ITGB2[38]. Another study reported that ITGB2 deficiency predisposes mice to osteoporosis, thus highlighting the connection between ITGB2 and bone development[39]. In summary, the microarray data provide deeper insight into the gene regulation of ACL-MSCs associated with the integrative healing processes required for repair in injured or diseased tissue while maintaining similar differentiation potentials.

This study had several limitations, including its cross-sectional design, which may be associated with a risk of reverse causality[22]. Samples from the patients who had undergone ACL reconstruction were damaged[8]. Although we attempted to obtain samples of intact ACL rather than from the rupture site, we could not entirely exclude the latter. Because of their rich vascularity, ruptured sites possess more stem cells than the mid-substance region of the ligament[17], which may affect the proportion of MSCs in freshly extracted samples (passage 0), especially in the ACL tear group. Furthermore, ACL samples from the TKA group may not be representative of the normal aging population without osteoarthritis[18].

Another limitation was the large female predominance in the TKA group. The female predominance in TKA has been observed universally across countries. However, this female predominant patient grouping may increase the multicollinearity, although the all VIF and Rj2 value for independent parameters in our study were relatively low (VIF<1.5, Rj2<0.3). Finally, we did not confirm that our marker criteria (CD34^−^/CD44^+^/CD90^+^) actually identified only MSCs; markers specific to MSCs have not been defined. However, we quantified MSCs as cells simultaneously satisfying the four surface marker criteria, whereas most previous studies of MSCs reported the percentage of each surface marker separately.

Despite these limitations, this study is the first to compare ACL-derived MSCs between young (ACL reconstruction group) and old (TKA group) patients. The results of the study showed that ACL-derived MSCs have similar differentiation potentials regardless of donor age. Given that ACL reconstruction and TKA are the most common operations performed around the world, our results suggest the possibility of using discarded ACL as a safe cell source for MSC production.

## Conclusions

In conclusion, the proportion of freshly isolated ACL-MSCs was higher in older patients with osteoarthritis than in younger patients with ACL tears, but their phenotypic and multilineage potential were comparable. Microarray data revealed differences at a genetic level between ACL-MSCs from old and young patients, indicating that differences in the regulation of genes, in particular those encoding ECM components, could be involved in the rate of tissue repair or regeneration modulated by MSCs in different age groups.
